# Hereditary Colorectal Cancer Syndromes: Small Bowel Cancer Risk and Endoscopic Surveillance Strategies

**DOI:** 10.3390/diagnostics15070819

**Published:** 2025-03-24

**Authors:** Edoardo Borsotti, Francesca Laura Nava, Felice Benedicenti, Laura Cini, Andrea Magarotto, Davide Ferrari, Paolo Cantù, Marco Vitellaro, Emanuele Rausa, Federica Cavalcoli

**Affiliations:** 1 Gastroenterology and Digestive Endoscopy Unit, Fondazione IRCCS Istituto Nazionale dei Tumori, Via Venezian 1, 20133 Milan, Italy; edoardo.borsotti@istitutotumori.mi.it (E.B.); laura.cini@istitutotumori.mi.it (L.C.); andrea.magarotto@istitutotumori.mi.it (A.M.); paolo.cantu@istitutotumori.mi.it (P.C.); federica.cavalcoli@istitutotumori.mi.it (F.C.); 2 Colorectal Surgery Unit, Fondazione IRCCS Istituto Nazionale dei Tumori, 20133 Milan, Italy; francescalaura.nava@istitutotumori.mi.it; 3 Unit of Hereditary Digestive Tract Tumors, Fondazione IRCCS Istituto Nazionale dei Tumori, Via Venezian 1, 20133 Milan, Italy; davide.ferrari@istitutotumori.mi.it (D.F.); marco.vitellaro@istitutotumori.mi.it (M.V.); emanuele.rausa@istitutotumori.mi.it (E.R.)

**Keywords:** familial adenomatous polyposis, hereditary colorectal cancer syndromes, small bowel cancer, surveillance, Lynch syndrome, capsule endoscopy, device-assisted enteroscopy, Peutz–Jeghers syndrome

## Abstract

**Background:** Hereditary colorectal cancer syndromes, including familial adenomatous polyposis (FAP), Lynch syndrome (LS), and Peutz–Jeghers syndrome (PJS), are associated with an increased risk of small bowel cancer (SBC). Due to the low incidence and non-specific presentation of SBC, effective surveillance strategies are essential for early detection and management. This review aims to evaluate and compare current endoscopic techniques for small bowel surveillance in these patients. **Methods:** A comprehensive review was conducted using peer-reviewed studies sourced from PubMed. Various endoscopic modalities, including capsule endoscopy (CE), device-assisted enteroscopy (DAE), and intraoperative enteroscopy (IOE), were assessed for their diagnostic yield, safety, and clinical utility. Surveillance recommendations of the different syndromes were also examined. **Results:** CE offers high sensitivity but lacks histological sampling capability. DAE, including double-balloon enteroscopy (DBE) and single-balloon enteroscopy (SBE), enables direct visualization, biopsy, and therapeutic interventions, albeit with greater procedural complexity. In FAP, duodenal surveillance follows the Spigelman classification to stratify cancer risk, while jejunal and ileal polyps remain less studied. LS patients have an increased SBC risk, warranting tailored endoscopic approaches. In PJS, surveillance aims to mitigate intussusception risks and allow early malignancy detection. **Conclusions:** Optimized surveillance strategies in hereditary colorectal cancer syndromes require a multimodal approach, integrating advanced endoscopic techniques with genetic risk stratification. Centralized care in tertiary centers improves outcomes by ensuring standardized surveillance protocols and enhancing early cancer detection. Artificial intelligence (AI) applied to CE and DAE is shaping promising prospects for the future surveillance of small bowel polyps by enhancing diagnostic accuracy and reducing the duration of the diagnostic process. Further research should investigate AI-assisted imaging and molecular biomarkers to optimize screening strategies.

## 1. Introduction

Small bowel cancer (SBC) is a rare malignancy in the general population, accounting for only 2.3% of all digestive cancers in the United States [1]. Nevertheless, the incidence of SBCs in the United States has increased over the past 20 years, rising from 5260 cases per year in 2004 to 12,440 cases in 2024, according to data from the American Cancer Society. Likewise, SBC-related mortality has also risen, from 1130 deaths in 2004 to 2090 in 2024 [2]. SBC comprises four main histological tumor types: adenocarcinomas, neuroendocrine tumors, stromal tumors, and lymphomas. Among these, small bowel adenocarcinomas and neuroendocrine tumors are the most common, each representing approximately 40% of cases, although the prevalence of subtypes may vary geographically [3,4].

SBC is a heterogeneous neoplasm with variable clinical behavior, influenced by factors such as tumor location, size, and histopathological features. It often presents insidiously, contributing to diagnostic delays [5]. A delayed diagnosis of small bowel cancer may significantly impact the subsequent therapeutic approach, possibly compromising the feasibility of curative treatment and, consequently, reducing overall and disease-free survival rates. The proximal small bowel (SB) is the most frequently affected site, with the duodenum involved in 55–82% of cases, the jejunum in 11–25%, and the ileum in 7–17%. Aparicio T. et al. reported a five-year survival rate for SBC ranging from 48% to 65%, and a median age at diagnosis within the sixth decade of life (55–64 years) [6].

Although SBC is rare in the general population, individuals with hereditary colorectal cancer syndromes face an elevated risk. These syndromes include inherited polyposis syndromes (such as familial adenomatous polyposis [FAP] and Peutz–Jeghers syndrome [PJS]), Lynch syndrome (LS), and constitutional mismatch repair deficiency (CMMR-D) [6,7].

In consideration of the poor survival for advanced stage, a timely and accurate diagnosis, as well as precise localization of SBC, are crucial. However, diagnosing SB lesions remains challenging due to their low incidence, non-specific clinical presentation, and anatomical location beyond the reach of standard endoscopic evaluation [8].

Since its approval in 2001, capsule endoscopy (CE) has become a key tool for SB evaluation, allowing a comprehensive inspection of the entire small intestine with a diagnostic yield of up to 91% for detecting SB lesions. Although CE is now the preferred first-line method for luminal SB assessment, its major limitation is the inability to obtain histological samples [9,10]. To overcome this, the introduction of device-assisted enteroscopy (DAE) has further expanded SB evaluation, offering a non-surgical endoscopic approach that enables direct visualization, tissue sampling, and therapeutic interventions within the SB [10].

### 1.1. Capsule Endoscopy

CE evaluation consists of a pill-size capsule that is swallowed and passes passively through the gastrointestinal tract via peristalsis. It can be administered in an outpatient setting and the device allows the evaluation of the SB without the use of ionizing radiation. Visualization of the entire SB is obtained in approximately 80% of patients [11]. CE is a well-tolerated procedure, and its safety profile is reported as excellent, with the major potential risk being capsule retention in 1–2% of cases due to postsurgical stricture, adhesion, or large polyps occluding the SB lumen [12]. However, the capsule retention rate varies among different studies in the case of SB tumors, and it may require endoscopic or surgical retrieval [13].

Nowadays, various types of endoscopic capsules are available on the market [14]. Despite their specific features, all share three main components: a capsule endoscope, a wireless data recorder, and a dedicated software for image review and interpretation [15,16].

Before administration, patients must undergo overnight fast and bowel preparation, typically with at least 2 L of a polyethylene glycol solution. Additionally, antifoaming agents such as simethicone are recommended to enhance visual clarity. After ingestion, the capsule passes through the gastrointestinal (GI) tract, propelled by natural peristalsis, without requiring lumen inflation.

Following capsule ingestion, patients have to observe a fasting period before gradually reintroducing clear liquids after 2 h and solid food after 4 h [15]. Meanwhile, a wireless device records images captured by the capsule’s cameras throughout the GI transit, which typically lasts between 8 and 12 h, depending on the capsule type.

Once the recording is complete, the video timeline is analyzed by an endoscopist using specialized software to generate an accurate report [15,16]. Automated software can assist in evaluating diffuse mucosal conditions, complementing conventional reading methods. To ensure comprehensive assessment, standardized scoring systems and transit-time indices are recommended for reporting findings and identifying lesions [17].

CE has been proposed in patients with hereditary colorectal cancer syndromes for SB inspection [18] and has shown an improved sensitivity over conventional radiological techniques for polyp surveillance [19,20,21] and a similar detection rate compared to DAE [22]. However, accumulating experience with CE combined with DAE and CT or MR imaging using enterography techniques has highlighted the potential limitations of CE, particularly in identifying solitary lesions, estimating the precise size and location of the lesions, and evaluating the papillary and duodenal region [19,23]. In addition, as stated before, CE does not provide the possibility to undergo tissue sampling or therapeutic procedures; for these reasons, in case of lesions observed at CE further endoscopic procedures are often required [16].

### 1.2. Device-Assisted Enteroscopy

DAE is a generic term for endoluminal examination of the SB by any endoscopic technique that includes assisted progression. It includes double-balloon enteroscopy (DBE), single-balloon enteroscopy (SBE), spiral enteroscopy, and balloon-guided endoscopy. Different from push enteroscopy (PE), which stretches the small bowel as the endoscope is advanced, most DAEs operate by sequential pleating of the small bowel over an endoscope and/or overtube. Pleating allows the endoscope tip to progress deep within the bowel avoiding excessive wall tension or looping. Compared to CE, DAE is a time-consuming technique which requires deep sedation but allows real-time-controlled observation with the option of tissue sampling and endoscopic treatment. DAE therapeutic options cover the whole range of widely used upper endoscopy and colonoscopy interventions.

#### 1.2.1. Double-Balloon Enteroscopy

The DBE systems allow deep intubation of the SB through the use of a high-resolution flexible enteroscope and a polyurethane overtube. The endoscope and overtube are both equipped with an inflatable latex balloon at the distal end. Each balloon is operated separately by a pressure-controlled inflation pump [24,25]. Currently, several DBE systems are available for different diagnostic and therapeutic indications. The DBE is advanced in the SB using repetitive cycles of alternating scope and overtube insertion, while manipulating the balloons to sequentially pleat the small bowel around the overtube. Complete small bowel visualization may generally be accomplished by a combined approach via the antegrade (oral) and retrograde (anal) route [17].

#### 1.2.2. Single-Balloon Enteroscopy

The SBE system was introduced nearly a decade after DBE as a more cost-effective and technically simpler alternative. SBE utilizes a similar overtube system but features only a single inflatable balloon, controlled by a pressure-regulated pump [26]. Unlike DBE, the enteroscope itself does not have a distal balloon.

While the simplified design enhances user-friendliness and broader adaptability, maintaining a stable position requires careful angulation of the enteroscope tip and suctioning against the small bowel wall. The procedural technique is similar to DBE, employing a “push-and-pull” maneuver to pleat the bowel onto the overtube.

#### 1.2.3. Spiral Enteroscopy

Spiral enteroscopy is characterized by an overtube that contains a soft raised helix (4.5 mm and 5.5 mm) at its distal end. Unlike balloon-assisted systems where the small bowel is pleated via the use of a balloon, the spiral enteroscopy systems advance through the small bowel via a “screw-like” mechanism.

The system can be used for both antegrade (Endo-Ease Discovery SB, Spirus Medical Inc., Stoughton, MA, USA) and retrograde small bowel enteroscopy (Endo-Ease Vista, Spirus Medical Inc., Stoughton, MA, USA). The overtube is mounted over an enteroscope, and the system is advanced into the SB [27,28]. The overtube has a coupling device that allows it to rotate at a fixed location on the enteroscope. Once mounted, the overtube does not move forward or backward in relation to the enteroscope. Movement within the small bowel results from physical clockwise rotation of the overtube and a “screw-like” pleating of the small bowel because of the raised helix [26]. During withdrawal, the endoscope is retracted to the 140 cm mark, followed by gentle counterclockwise rotation to safely extract the device [17].

Recently, motorized spiral enteroscopy (MSE) or PowerSpiral enteroscopy (Olympus Medical Systems, Tokyo, Japan) were introduced as advanced techniques in the field of enteroscopy. MSE is a novel concept with a self-propulsive motorized enteroscope that pulls the bowel toward the MSE by rotation of the spiral overtube, allowing faster procedure time and achieving deeper insertion compared to traditional spiral enteroscopy [29,30]. However, unfortunately, despite expectations, several major adverse associated events were noted, including deaths, and the method was withdrawn by the manufacturing company.

### 1.3. Intraoperative Enteroscopy

Intraoperative enteroscopy (IOE) consists in the direct exploration of the SB using a flexible endoscope during a surgical procedure. The endoscope can be introduced either orally or through an enterotomy. Its progression through the bowel is facilitated by manual external manipulation by the surgeon, ensuring thorough visualization and, if needed, therapeutic intervention [17].

The main features of different enteroscopy modalities are summarized in Table 1.

### 1.4. Artificial Intelligence Applied to SB Endoscopy

In the last decade, the introduction of artificial intelligence (AI) technologies applied to endoscopic procedures further expanded the potential of endoscopic surveillance and assessment of small bowel diseases; these technologies are aimed at improving diagnostic accuracy, in terms of lesion detection and classification, giving real-time support to clinicians in optimizing clinical workflows [34].

AI applications employing CE and enteroscopy have shown promising results in implementing the accuracy and shortening the duration of the diagnostic procedures, although the studies evaluating this tool mainly refer to the general population and not specifically to the surveillance in hereditary colorectal cancer syndromes [35,36]. Both investigations display high levels of sensitivity and specificity, indicating their effectiveness in the detection of mucosal lesions overlooked by the human eye. Interestingly, in most studies, AI demonstrated a better proficiency in recognizing true negatives over true positives, thus improving specificity over sensitivity rates [34]. SB investigation is often associated with the presence of artifacts such as bubbles and luminal debris, united with intense peristalsis and motility. These conditions introduce challenges in image interpretation and application of processing capabilities, requiring both algorithmic refinement and optimized hardware solutions [37]. Furthermore, considering that AI algorithms rely on extensive training datasets, the relatively small number of SB lesion series may hamper algorithm development and generalizability and produce biased results [34,37].

In conclusion, AI applied to enteroscopy raises expectations for the future of diagnosis and surveillance of SB polyps, improving diagnostic accuracy and providing the basis for better definition of more sensitive and specific algorithms in this setting of patients. To date, the creation of accurate and generalizable AI models remains challenging and will require further efforts and investigations in order to routinely integrate the full potential of AI in the management of SB diseases.

## 2. Materials and Methods

The literature search focused on peer-reviewed studies published in English, primarily sourced from PubMed. The following Medical Subject Headings (MeSH) were entered into the database search field: “endoscopic techniques”, “small bowel”, “small bowel cancer” “small bowel neoplasm”, “endoscopic surveillance”, “hereditary colorectal cancer syndromes”, “Lynch Syndrome”, “familial adenomatous polyposis”, “Peutz-Jeghers syndrome”, “capsule endoscopy”, “device-assisted enteroscopy”, “intraoperative enteroscopy”, “double-balloon enteroscopy”, “single-balloon enteroscopy”. A comprehensive screening process was then employed, starting with title and abstract screening to exclude irrelevant studies, followed by a comprehensive full-text review to identify those meeting the inclusion criteria.

This review prioritizes studies with high levels of evidence, including randomized controlled trials (RCTs), systematic reviews, and cohort studies with sufficient sample sizes, all published in peer-reviewed journals. In cases where experimental data were unavailable, relevant observational studies were also considered. Recent research was emphasized, particularly in areas with significant advancements; however, older studies were included when they provided essential foundational knowledge.

## 3. Familial Adenomatous Polyposis

FAP is an autosomal dominant inherited disorder caused by a germline mutation in the adenomatous polyposis coli (*APC*) gene, a tumor suppressor gene, located on the long arm of chromosome 5 (5q21-22) [38]. The reported incidence at birth is about 1/8300, equally in both sexes, and accounts for less than 1% of colorectal cancer (CRC) cases [39,40]. Although most patients are identified at a young age due to a positive family history of FAP, a quarter of patients have a de novo *APC* mutation, presenting with symptoms at an advanced stage.

The disease is characterized by the formation of hundreds to thousands of colorectal adenomas, typically arising at teenage years, following an anticipated, multifocal adenoma–carcinoma sequence compared to sporadic adenomas. The risk of developing CRC approaches 100% at a young age with a median of 35–45 years [41]: therefore, prophylactic proctocolectomy and an ileoanal pouch or total colectomy with ileorectal anastomosis are mandatory around the second or third decade of age.

However, life expectancy of FAP patients remains lower than that observed in the general population, due to the development of upper-GI polyps, extraintestinal manifestations, and desmoid tumors (DTs) [42].

Although the colon and the rectum are the most commonly involved regions, nearly all patients with FAP may develop gastric fundic gland polyps, gastric adenomas, duodenal adenomas, and carcinomas.

The duodenum is the second most common site of polyp development, reporting a prevalence between 30% and 92% of FAP individuals with a lifetime risk approaching 100%; the lifetime risk of progression to duodenal cancer is estimated about 4–12% and represents the leading cause of death in FAP [42,43]. Duodenal adenomas tend to occur approximately 15 years after the appearance of colonic adenomas; thus, endoscopic surveillance of the upper gastrointestinal tract is pivotal in an effort to prevent duodenal surgery as well as duodenal cancer.

While duodenal polyps are incorporated into standard surveillance guidelines, the significance and management of polyps in the jejunum and ileum have not been established yet. Non-duodenal SB adenomas (beyond the ligament of Treitz) are less common than duodenal adenomas, with a prevalence of jejunal and ileal adenomas ranging from 45% to 75% and 10% to 20%, respectively [44]. Although there are no standardized strategies to treat jejunal and ileal polyps in patients with FAP, these SB polyps have the potential to develop into cancer via the adenoma–carcinoma sequence [45].

### 3.1. Duodenal Surveillance

Guidelines recommend complementing standard forward-viewing duodenoscopy with a side-viewing duodenoscope or with cap-assisted forward-viewing endoscopy in patients with FAP, in order to better evaluate the ampulla, as it is well demonstrated that approximately 50% of duodenal cancers are located in this region. Life-long endoscopic surveillance is recommended from age 20–25 years in FAP, and is guided by Spigelman stage (Table 2) and papillary endoscopic appearance.

For these reasons, the current consensus guidelines recommend [46,47,48] the following:

Initial Surveillance: starting in late teens/early 20s, within two years of FAP diagnosis;
Spigelman stage 0, I: Repeat EGD every 3–5 years;
Spigelman stage II: Repeat EGD every 2–3 years;
Spigelman stage III: Repeat EGD every year;
Spigelman stage IV: Repeat EGD every 3–6 months, consider advanced therapeutic or surgical interventions.

This staging system has been validated by different studies and stratifies the risk of developing duodenal carcinoma according to the number, size, histology, and degree of dysplasia of the polyps (Table 3).

**Figure 1:**
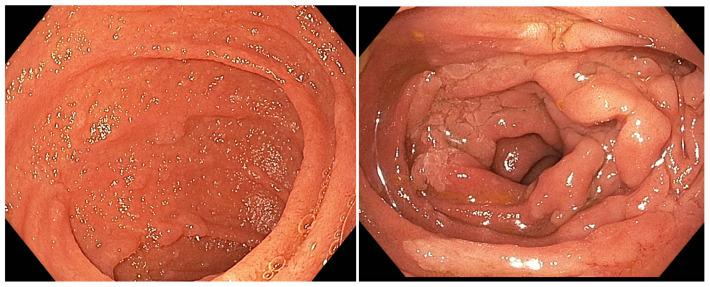
Endoscopic images of duodenal adenomas at different Spigelman stages: Stage I on the left and Stage IV on the right.

Based on this system, approximately 70% to 80% of patients with FAP have stage II or III duodenal disease and 20% to 30% have stage I or IV disease [49,50]. Figure 1 depicts images of patients with Spigelman stage I and IV duodenal adenoma.

While the Spigelman classification continues to be a valuable tool for staging non-ampullary adenomas, its predictive power and role in guiding management may present some limitations [48,51]. The first is the need for histopathologic sampling, although nowadays it is well known that routine biopsies of duodenal polyps may interfere with optical diagnosis and future endoscopic resection, and they are currently not recommended by guidelines. Moreover, it should be noted that the Spigelman classification alone lacks information on the (peri-) ampullary site, which is the most common site of development of duodenal carcinoma [48].

**Figure 2:**
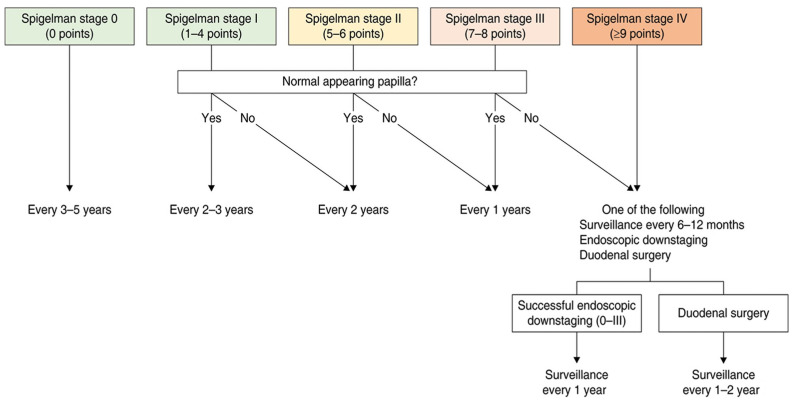
Surveillance intervals according to duodenal and papillary findings as proposed by the last updated European guidelines [48].

Therefore, recent studies emphasize the wide variability in polyp progression and malignancy risk within each Spigelman stage, suggesting the need for a more personalized approach and the inclusion of additional risk factors for papillary cancer (Figure 2) [51,52,53,54].

The macroscopic appearance of duodenal adenomas in patients with FAP varies widely: these lesions are usually white and numerous and may present as flat sessile polyps, nodules or subtle abnormalities of the mucosa, and can be located either in the duodenal bulb, ampullary/periampullary region, or distal duodenum [38,42]. Smaller adenomas may easily be missed during upper endoscopy.

Although biopsy samples of non-ampullary polyps are discouraged, performing random biopsies of macroscopically normal papillae may enhance the detection rate of adenomas. Macroscopically regular ampullas may present abnormal histology in up to 44% of cases, including low-grade dysplasia (8–25%) and high-grade dysplasia (<0.5%) [48,52].

Virtual chromoendoscopy techniques such as narrow-band imaging (NBI) or dye-spray chromoendoscopy have been proposed as a tool for improving the diagnostic yield, leading to an upstaging of the Spigelman stage but without known clinical outcomes [55,56]. Several studies suggest endoscopic characterization to differentiate low-grade from high-grade duodenal adenomas based on the pit pattern, vascular pattern, presence and distribution of the white opaque substance, and color and size of the lesions [57], although no standardized classification (such as the Kudo Classification applied to colorectal polyps) has been proposed.

Other studies are evaluating the role of advanced endoscopic imaging techniques, such as confocal laser endomicroscopy, in order to better characterize dysplasia and improve detection of early malignancy. These techniques may help with more targeted biopsies and surveillance intervals.

**Figure 3:**
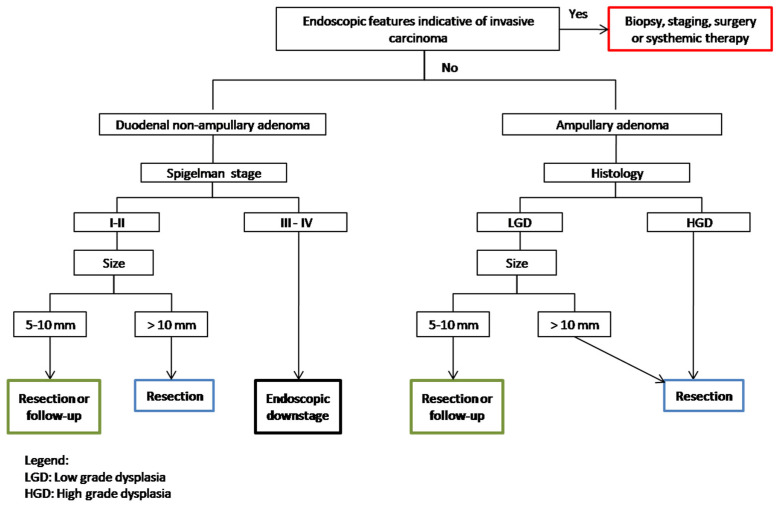
Management algorithm for the treatment of duodenal adenomas in FAP.

Although surgery is considered a conclusive treatment and the gold-standard treatment for FAP patients with severe polyposis (stage IV), in less advanced cases, depending on the size and number of the polyps, endoscopic resection may be effective in preventing the development of duodenal cancer and in deferring duodenal surgery. Management strategies are evolving, with greater emphasis on personalized therapy. Patients with advanced duodenal polyposis (Spigelman stages III or IV) or advanced papillary lesions should undergo endoscopic downstaging; this practice may reduce the risk of progression to cancer, even though the risk of re-progressing to stage IV within 1 year is reported in 50% of cases [53,54,58]. Clinical practice and cohort studies suggest that duodenal non-ampullary polyps >10 mm should be removed (Figure 3), as lesions <10 mm rarely contain HGD or invasive carcinoma [53,58].

The current therapeutic approaches are:

- Endoscopic polypectomy: depending on the features and dimensions of the polys; cold snare polypectomy for small lesions (<6 mm in size) and endoscopic mucosal resection (EMR) for larger lesions are the first-line endoscopic resection techniques for non-malignant non-ampullary duodenal adenomas. These techniques have been demonstrated as being safe, with good success rates and an acceptable adverse event profile [59]. In recent retrospective studies, high rates of complete endoscopic resection (90.5–96.1%) have been described, whereas the adverse event rates ranged from 2% to 24.4% [59,60]. In the last decades, different studies reported promising outcomes on emerging endoscopic resection modalities, such as underwater EMR and piecemeal cold snare EMR for the treatment of duodenal polyps, reporting a higher en bloc resection rate and lower rate of adverse events [61,62,63,64,65,66].

Endoscopic Submucosal Dissection (ESD): although less commonly used, ESD is being explored for selected indications (suspected superficial submucosal invasion, non-lifting lesions due to de novo submucosal fibrosis, or secondary to previous incomplete resection), offering the potential for en bloc resection. Nevertheless, considering the higher incidence rate of duodenal perforation/bleeding, reaching 15–25% in some studies, and the lack of significant differences in long-term outcomes and survival compared to patients treated with EMR, ESD should be considered to be accurate in expert ESD centers [59,67].

### 3.2. Periampullary Lesions

The management of periampullary lesions follows a more complex algorithm. Accurate preoperative diagnosis and staging of ampullary adenomatous lesions are critical for predicting prognosis and determining the most appropriate therapeutic approach. To date, there is no widely accepted classification system for ampullary adenomas. Granular or villiform exophytic lesions are most commonly encountered and associated with a favorable histology. These lesions may present a laterally spreading component, extending onto the duodenal wall beyond the papilla. Smooth elevated lesions are less frequent but are associated with a greater risk for invasive disease [68,69]. Endoscopic features suggestive for invasive carcinoma include ulceration, infiltrative border, and a hard consistency [65,70]; such endoscopic findings should raise the suspicion of invasive carcinoma, discouraging an endoscopic approach for the treatment [58].

To prevent ampullary cancer, endoscopic papillectomy should be considered in patients showing adenomatous changes in the ampulla >10 mm, associated with high-grade dysplasia or villous histology (Figure 2). Linear and circular EUS are widely used in the staging and assessment of ampullary lesions, especially for the evaluation of the depth of mucosal invasion; nevertheless, there is no consensus regarding whether EUS should be used as a routine examination before papillectomy [48,58]. Magnetic resonance cholangiopancreatography is an alternative non-invasive imaging technique that can also evaluate pancreaticobiliary conditions and intraductal invasion. Additionally, ERCP plays an essential role in pretreatment staging of ampullary adenomas, especially in determining intraductal extension; ERCP plays also a role in reducing the risk of post-papillectomy pancreatitis and in the management of obstructive jaundice in ampullary adenomas [68].

Endoscopic papillectomy (EP) is performed using a standard duodenoscope; several endoscopic resection techniques have been established for the lesions of the papilla, including snare polypectomy, EMR, and endoscopic submucosal dissection [59,68,71,72]. Recent studies suggest that a simple papillectomy without a submucosal epinephrine injection may be recommended for patients with ampullary adenomas, as it is associated with lower adverse events and lower recurrence rates [59,73,74]. However, papillectomy is associated with severe complications, with an overall rate ranging from 7.7% to 58.3%, such as pancreatitis, bleeding, perforation, papillary cholangitis, and papillary and duodenal luminal stenosis [75]. Experts suggest prophylactic pancreatic stent placement in order to decrease the risk of post-EP pancreatitis and late post-procedure stenosis, especially in cases with insufficient pancreatic juice drainage after EP [68,71]. Complete endoscopic resection is reported in 63% of cases to show worse R0 rates compared to non-FAP patients, thus underlining the importance of an accurate long-time surveillance [76].

Surgical Resection: pancreas-sparing duodenectomy and pancreato-duodenectomy are reserved for high-grade dysplasia, advanced lesions, or lesions not amenable to endoscopic treatment; both procedures present similar safety and efficacy outcomes, including 10-year overall and disease-specific survival, 30-day mortality rate, and morbidity rate [77,78]. After duodenal surgery, surveillance of the jejunum should be considered, especially in patients with stage III/IV polyposis, even though evidences regarding the prevalence of jejunal polyps are poor and a correct post-surgical management should be assessed in a research setting [79,80].

### 3.3. Jejunal/Ileal Surveillance

Non-duodenal SB polyps have been reported in 43–87% of cases, described mainly as diminutive polyps and localized in the jejunum. However, different cases of advanced adenomas (>10 mm; high-grade dysplasia) have been diagnosed in the distal duodenum and jejunum, leading to an increased risk of malignancy [81]. According to FAP registry studies, non-duodenal SB cancer is reported at a frequency of 8.5% in the jejunum and 1.7% in the ileum, with an estimated risk which is more than 100-fold the risk of the general population. To date, international guidelines do not recommend small bowel surveillance for non-duodenal polyps in FAP patients due to poor data and low-quality evidence [82]. Previous studies suggested that FAP patients with more severe Spigelman score have a higher risk of developing SB polyps [83]. Indeed, such polyps occur frequently in patients who underwent previous duodenectomy due to advanced duodenal polyposis [84,85,86]. For these reasons, SB surveillance has been proposed for FAP patients with a higher Spigelman stage. In the last decades, the development of CE and DAE has enabled direct visualization of the SB, creating new strategies for adenoma surveillance in FAP patients.

In regard to CE, a few studies have investigated its feasibility and sensitivity in patients with FAP, demonstrating an increase in the diagnostic yield of SB tumors as compared to EGD [70,87,88,89,90,91,92,93]. Most series observed a significant correlation between the number of duodenal adenomas or Spigelman stage and the number of jejunal/ileal polyps detected at CE.

On the other hand, data on a possible correlation between the higher Spigelman stage and the risk on onset of SB cancer are scanty and non-conclusive, also due to the relatively low rate of incidence of these neoplasms [81,94,95]. Furthermore, although CE offers a non-invasive complete and panoramic exploration of the SB, this endoscopic technique is limited by the impossibility of conducting a biopsy or resecting non-duodenal SB lesions. The reported safety profile for CE was excellent, with very limited adverse events, including CE retention, such as the CE retention reported in the published literature.

Accordingly, a Japanese prospective study aimed at evaluating the safety of CE in 41 FAP patients who underwent previous colectomy observed no adverse events. The authors reported an incidence of non-duodenal SB polyps in 51% of FAP patients, including a case of intramucosal adenocarcinoma [96]. Recently, Fukushi et.al. in a retrospective study on 64 FAP patients who underwent CE reported a significantly higher prevalence of small-intestinal tumors for patients in Spigelman stage III and IV groups than those in the stage 0 group (*p* < 0.05). In addition, the author showed the presence of pathogenic variants in the *APC* codons 278 and 1062–1504 to be a significant independent risk factor [44]. However, all the studies showed a limitation of CE in the evaluation of the duodenum and the periampullary area, where the majority of small bowel cancers occur, and poor reproducibility in the estimation of polyp size and localization [84,85,86].

Currently, few studies compared the outcomes of CE with radiologic techniques for SB surveillance. In 2004, a study of 20 patients with FAP reported CE to be superior to MRI-E and SB follow-through for the identification of polyps in the jejunum and ileum. However, MRI-E has been shown to be more accurate than CE in locating larger polyps and in determining their exact size [19].

DAE may be an alternative in the surveillance of advanced FAP patients, allowing a more accurate localization and characterization of the lesions and offering the possibility of resection or sampling of advanced lesions. Only a few studies are available on the use of DAE in FAP [97,98,99]; these case series reported the occurrence of adenomas predominantly in the jejunum and in most cases SB polyps were diminutive, while no adenocarcinomas were detected. As previously reported, these studies suggested a significant association with the severity of duodenal polyposis. In another recent Italian multicenter study, aimed at evaluating the diagnostic and therapeutic yield of SBE in patients with suspected SB disease (detected with non-invasive methods), in 5 of 7 FAP patients DAE was found to be diagnostic and allowed an endoscopic resection in 3 cases. Nevertheless, it should be noted that these endoscopic procedures are quite invasive, and require long endoscopic times and an inpatient setting. Thus, DAE should be considered for selected patients with high suspicion of SB disease. Different studies showed DAE to be a safe procedure with no significant adverse event rates [82,97,98,99].

### 3.4. Insights on Clinical Practice

The current guidelines generally focus on surveillance of the duodenum, with less definitive recommendations for the evaluation of non-duodenal SB polyps in FAP patients.

For duodenal surveillance in FAP patients, standard forward-viewing duodenoscopy, either with a side-viewing duodenoscope or cap-assisted forward-viewing endoscopy (for ampullary region evaluation), is recommended starting at age 20–25 years. Surveillance is guided by Spigelman stage and papillary appearance, as summarized in Figure 2. The use of virtual chromoendoscopy might be suggested to enhance adenoma detection and improve polyps’ characterization.

Endoscopic resection of non-ampullary adenomas >5 mm is advised to prevent Spigelman stage progression. Based on the current data, aggressive endoscopic resection might be beneficial for patients with Spigelman stage III and selected stage IV cases to reduce adenomatous involvement and lower the risk of cancer progression.

Endoscopic papillectomy should be considered for patients with ampullary adenomas >10 mm, high-grade dysplasia, or villous histology.

The primary goal of SB surveillance in FAP patients is to prevent SBC by identifying and endoscopically removing jejunal and ileal pre-malignant adenomas or early cancerous lesions. Currently, ASGE guidelines restrict SB enteroscopy to grade IV Spigelman patients [100], while ESGE guidelines suggest the use of capsule endoscopy (CE) and/or cross-sectional imaging to detect SB polyps [16].

Nevertheless, since previous studies have shown a significant correlation between Spigelman stage and the number of jejunal/ileal polyps, we recommend push enteroscopy for all patients with Spigelman stages III and IV. In cases of a high polyp burden in the jejunum or evidence of adenomas beyond the reach of push enteroscopy, DAE should be scheduled for a more comprehensive SB evaluation. Currently, there is lack of consensus on the timing of a follow-up procedure for SB surveillance; in the presence of a high polyp burden, we suggest scheduling DAE at 3–6 months, while a 12-month interval appears reasonable for minimal residual disease.

In addition, enteroscopy should be considered in FAP patients using a multidisciplinary approach and tailored to patients presenting with symptoms, or in the setting of preoperative screening in patients awaiting duodenal surgery.

Further studies should be addressed at identifying the individual risk factors and specific pathogenetic variants connected to the development of non-duodenal SB lesions and at evaluating the long-term outcomes of these patients.

## 4. Lynch Syndrome

LS, also known as hereditary non-polyposis colorectal cancer (HNPCC), OMIM #120435, is an autosomal dominant genetic disorder linked to the germline mutation of a DNA mismatch repair (MMR) gene (*MLH1*, *MSH2*, *MSH6*, *PMS2*) or to a deletion in the 3′ region of the epithelial cell adhesion molecule (*EPCAM*) gene 4. These mutations cause errors during the DNA replication process, leading to microsatellite instability (MSI) [101]. In this context, *MLH1* and *MSH2* mutations are the most frequent, accounting for 80–90% of all LS cases [102]. In clinical practice, LS is associated with an increased risk of malignancies in multiple organs, including the colorectum, endometrium, stomach, ovary, pancreas, small intestine, renal pelvis, biliary tract, and brain [103]. LS is typically suspected in individuals with early-onset gastrointestinal, endometrial, and urothelial cancers. Additionally, the Bethesda revised [104] and Amsterdam II criteria [105] may be utilized to select individuals at high risk for LS. In these cases, a genetic evaluation should be recommended and a specific genetic test carried out. Genetic testing is usually conducted on blood or saliva of patients over 18 years old to identify mutations in the following MMR genes: *MLH1*, *MSH2*, *MSH6*, PMS2, and *EPCAM*. Additionally, BRAF mutations and the methylation of the *MLH1* promoter are also investigated, considering that these conditions may mimic the LS presentation [106]. Before testing, genetic counseling is usually advised in order to discuss the implications of the potential results and provide information on the hereditary patterns. If one of the mutations is confirmed, a second genetic counsel usually occurs, to identify the patient’s needs and guide future surveillance decisions for the patient and their family [106]. More recently, immunohistochemistry (IHC) application for all newly diagnosed colorectal cancers has been becoming more and more common in clinical practice and may further help in suspecting a genetic disorder.

The reported lifetime risk of SBC in LS patients ranges from 0.4% to 12%, leading to a relative risk more than 100 times higher than that of the general population [106,107,108,109,110,111]. In 2007, a retrospective study based on the Dutch HNPCC registry reported an absolute lifetime risk of SBC in LS patients of 4.2%. The investigated risk factors, such as sex, family history, and previous CRC history, do not seem to significantly increase the lifetime risk [107]. Interestingly, almost 50% of SBCs were located in the duodenum, with a decreasing frequency progressing from the duodenum to the ileum. Specifically, considering all described SBCs in LS, 43% of them were located in the duodenum, 37% in the jejunum, and 20% in the ileum [89]. In LS, SBC develops along the adenoma–adenocarcinoma sequence, as in CRC, and duodenal adenomas have been reported in 1.5–2.4% of the patients in Western countries [86].

Møller et al. investigated the different cancer presentations depending on age and pathological variants. In fact, the different mutations are associated with different cumulative risks of cancer development. For example, pathological variants of *MLH1* determine a relative cumulative incidence of SBC at 75 years of 64.7%, while *MSH2* variants are associated with a 20.1% risk and *MSH6* and *PMS2* carry none. The median age at diagnosis of SB is between 39 years and 53 years, about 10–20 years earlier than the general population, with a cumulative incidence of 0.4% at 40 years and 6.5% at 75 for the *MLH1* mutation [112].

Notably, surveillance for SB neoplasms in LS patients is still a subject of ongoing discussion. To date, given the low incidence of SB cancer and the fact that most cases occur in the duodenum or distal ileum (areas accessible during routine gastroscopies and colonoscopies), endoscopic screening of the jejunum and ileum is generally not recommended [106,111,113]. However, it could be considered in the presence of a risk factor such as *MLH1* pathogenic variant mutation. The recommendations on when a SB surveillance should be initiated diverge widely; some authors do not suggest surveillance, whereas others advise that 30–40 years should be the age target [114].

### 4.1. Diagnostic Modalities

Over time, different methods to investigate the SB via surveillance in LS patients have been proposed. Conventional endoscopic techniques, such as gastroduodenoscopy, push enteroscopy, and ileo-colonoscopy, hardly allow for the whole exploration of the SB [115]. However, since most lesions occur in the proximal upper gastrointestinal tract, specifically in the duodenum, standard push enteroscopy have been reported to be able to detect a significant number of lesions [116]. Recently, the accessibility of the entire SB has then been increased with the introduction of newer techniques, such as CE and DAE [89].

#### 4.1.1. Capsule Endoscopy

CE has been reported as a safe, well-tolerated procedure in LS, with a rate of reported complication ranging from 0 to 0.5% [109,117,118]. The diagnostic yield of CE in LS patients is still under investigation, with studies reporting an incidence as high as 8.6% of asymptomatic SB neoplasms [108], others reporting 1.5% of affected patients, with lesions located within reach of surveillance gastroscopies [109], and some others describing a 4.4% incidence of neoplasms [117]. However, the CE-described lesions have no histological confirmation, so patients need to undergo a more invasive procedure for diagnosis, such as DBE or surgical evaluation. Furthermore, it needs to be considered that both false negative and false positive results have been described for CE in this setting. The main outcome data for CE in LS are presented in Table 4.

A recent meta-analysis investigated the CE surveillance in 428 LS patients. At the first-round screening, the estimated diagnostic yield of CE in identifying possible pathological findings was 8% (95% CI 4–12%), with this result lowering to 2% after pathological confirmation. For second-round screening at approximately two years, the estimated pooled yield was 6% (95% CI 2–10%) for CE-identified pathology, with a decrease to 0% for histologically confirmed lesions. Thus, the authors concluded against the routine use of CE for SB cancer screening in asymptomatic LS individuals [112].

Interestingly, Haanstra et al. showed that CE surveillance with a mean of 2.2 (range 1–6) years helped to identify possible significant lesions in 17 of the 155 (11%) patients. These 17 lesions required further investigations: gastroduodenoscopy in 8 patients and DBE in 9 [118].

Furthermore, Perrod et al. [117] corroborated the aforementioned results showing that after a 2-year surveillance period, new lesions arose in 4.6% of patients who were negative at baseline. Therefore, this surveillance interval may be warranted, particularly for patients with higher-risk mutations.

#### 4.1.2. Device-Assisted Enteroscopy

DAE has been reported in different series after CE for direct identification of possible mucosal alterations [109]. This procedure is reliable in exploration of the SB and allows direct access to the lesions, offering samples for histological determination. It also allows for mucosal tattooing in cases where precise localization of the lesion is required [119]. However, DAE is time-consuming and invasive, often requiring both oral and anal access. Therefore, in consideration of the low risk of tumor development and the frequent localization in the duodenum, the current use of DAE as a screening tool in LS is not routinely recommended [107].

### 4.2. Insights on Clinical Practice

Surveillance for SB lesions in LS patients is still debated in the literature. The poor survival rate (67% at 5 years [120]) of LS patients with SBC could indicate the need to invest in early detection.

Nevertheless, to date, the low incidence of SB lesions in LS does not justify routine SB examinations in this context. However, previous studies have reported a significantly higher cumulative incidence of SBC in patients with pathogenic variants of *MLH1* and *MSH2*, making SB surveillance with CE a reasonable approach in these individuals.

In cases of suspected findings on CE, DAE should be performed to confirm the presence of lesions, obtain a pathological diagnosis, and facilitate either endoscopic treatment or mucosal tattooing to optimize further surgical approaches.

Currently, data on the appropriate age for initiating SB surveillance and the recommended timing for follow-up evaluations are lacking. However, considering that the median age at SBC diagnosis in LS patients ranges between 39 and 53 years, initiating SB surveillance between the ages of 35 and 40 appears reasonable, with follow-up procedures scheduled every 2 to 4 years.

For patients presenting with abdominal pain or unexplained anemia, MRI-E or CE should be the first-line diagnostic modality.

Further multicenter studies involving larger LS cohorts are needed to better identify potential risk factors for SBC and to determine which patients would benefit most from SB surveillance.

## 5. Peutz–Jeghers Syndrome

PJS is a hereditary autosomal dominant genetic polyposis syndrome, with incomplete penetrance. It is caused by a germline mutation, found in 80–94% of PJS patients, in the tumor suppressor gene serine/threonine kinase 11 (*STK11*), located on chromosome 19p13.3, which encodes the liver kinase B1 protein (LKB1) [121].

However, 6–28% of patients with PJS have negative genetic tests, likely due to unidentified gene loci or mosaicism. Additionally, up to 20% of PJS patients have no family history, as 45% of cases arise from de novo mutations. PJS is a relatively rare disease, with an estimated incidence of 1:50.000–1:200.000 births and a prevalence of 1:200.000 people [122].

The main features of Peutz–Jeghers Syndrome include multiple GI hamartomatous polyps and distinctive mucocutaneous pigmentation.

The mucocutaneous pigmentations are small flat dark-brown oval macules typically located in oral mucosa, nostrils, and lips, arising during childhood. These lesions are observed in almost 90–95% of PJS patients [123,124].

Hamartomatous polyps usually appear during early childhood or puberty. Polyps may be sessile or pedunculated, varying in number, from one to hundreds, and size range, from a few millimeters to several centimeters [125,126]. They show typical histopathological features, being composed by normal cells of the GI tract but presenting a distorted architecture due to the proliferation of muscle cells of muscularis mucosae [127]. The most involved site is the SB (60–90%), particularly the jejunum and ileum, followed by the large bowel (50–64%) and stomach (15–30%), the while esophagus is not involved.

Furthermore, these patients are at increased risk for GI and extra-GI cancers (breast, ovarian, uterine, testicular) [128,129]. Among GI cancers, the lifetime risk is 36–39% for colorectal, 24–29% for gastric, 11–36% for pancreatic, and 10–14% for SB cancer [130], while the cumulative risk of all gastrointestinal cancers (except for pancreatic cancer) has been estimated to be 57% at 70 years old [131]. Although the pathophysiology of GI cancer related to Peutz–Jeghers disease is still unknown, a hamartoma–adenoma–carcinoma pattern has been suggested [131].

The most common clinical manifestations are iron-deficiency anemia, due to occult blood loss, or overt gastrointestinal bleeding (melena or hematochezia, according to the site of GI bleeding), while the most important complication is bowel sub-obstruction or obstruction caused by intussusceptions [82].

The natural history of the SB disease consists of polyps’ growth causing intussusceptions, followed by surgery and consequent risk of intra-abdominal adhesions and short bowel syndrome with intestinal failure [132]. Indeed, these patients have a high cumulative risk of SB intussusception at young age (50% by the age of 20 years), often requiring emergency surgery. Polyp size is the most important feature affecting the risk of intussusception, and previous studies have demonstrated that polyps with a diameter ≥15–20 mm are at increased risk [16,133,134].

Hamartomas are the most representative histotype of polyps found in PJS; however, other histotypes, such as adenomatous, hyperplastic, and inflammatory polyps, may also be found, although less frequently. Therefore, distinguishing between hamartomatous and adenomatous polyp is crucial, given the higher risk of malignant progression associated with the latter, and the consequent need for adenoma removal regardless of polyp size.

The rationale of SB surveillance in PJS patients is to detect and then remove endoscopically large polyps, thus reducing polyp burden and risk of complications and avoiding emergency surgery [135]. Another main purpose, particularly in advanced age, is the SB cancer screening aimed at detecting premalignant or malignant lesions at an early stage [131]. In fact, the detection and subsequent removal of large polyps would potentially decrease the risk of SB cancer, even if the efficacy of SB surveillance for cancer screening has not been evaluated in controlled studies [16].

Traditionally, small bowel follow-through and barium enteroclysis have been used for small bowel surveillance of patients with PJS, but there is increasing concern regarding the risk of cumulative radiation exposure associated with these tests, especially in children and young patients. Therefore, nowadays, CE and magnetic resonance enterography (MRI-E), are the two most used methods [136].

The current European (ESGE, EHTG) and American (ACG, AGA) guidelines recommend a baseline SB evaluation at the age of 8 years in asymptomatic patients affected by PJS, and earlier in symptomatic patients. In case of polyps’ detection during the baseline procedure, the recommended interval of SB surveillance is 1–3 years, based on the polyps’ phenotype (size, number, site), while, in case of negative baseline endoscopy, routine SB surveillance should begin at the age of 18, with the same interval range. Elective polypectomy is indicated for small bowel polyps ≥15 mm in diameter or for symptomatic smaller polyps [47,133,137,138].

Depending on local availability and expertise and patient preference, CE or MRI-E are both recommended for SB surveillance, according to European and AGA guidelines, whereas ACG guidelines recommend CE (Table 5).

### 5.1. Diagnostic Modalities

#### 5.1.1. Capsule Endoscopy

CE has been demonstrated to be a safe, well-tolerated, and feasible procedure in PJS [21]. Previous studies demonstrated the higher sensitivity of CE over conventional radiological procedures and similar detection rate of SB polyps compared to MRI-E [20,21,86,139,140].

In 2014, Urquhart et al. [140] compared CE and MRI-E in 20 patients who underwent both investigations and DBE confirmation, reporting a higher rate of patients with at least one significant polyp identified by CE compared to MRI-E (55% vs. 35%; *p* = 0.25).

More recently, Gupta et al. [139] did not observe a significant difference between the two modalities in detecting clinically relevant polyps (>10 mm), even if, in three patients, large polyps (>15 mm) detected via MRI-E were not identified via CE. Additionally, the authors observed significant discrepancies in polyp size measurement between the CE and the resected specimens. Overall, this study reported that CE was significantly more comfortable than MRI-E (*p* = 0.002), with no cases of capsule retention.

Consistent with this study, clinically relevant SB lesions can be missed during CE, notably in cases of isolated lesions located within the proximal small intestine (duodenum and proximal jejunum), probably due to the rapid capsule transit in the first tertile, bile and bubble artifacts, and less luminal distension [141,142].

**Figure 4:**
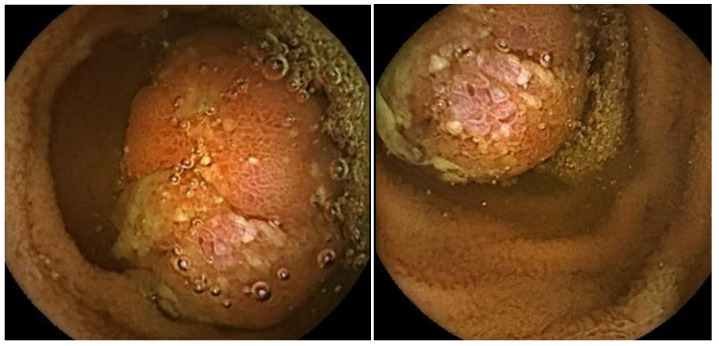
Jejunal hamartomatous polyp detected at CE in a patient affected by PJS.

Ultimately, based on the current literature, both CE and MRI-E are reasonable options for small bowel surveillance in PJS, with a similar detection rate for polyps having a diameter between 10 and 15 mm, while a higher sensitivity of CE has been reported for the detection of small polyps (<10 mm), and a higher accuracy of MRI-E for larger PJS polyps (≥15 mm) and for the assessment of SB polyps’ size and site. Of note, CE showed an exceedingly high safety profile, and it has been reported to be better tolerated by patients than MRI-E. Figure 4 depicts an exemplificative image of a hamartomatous polyp in PJS detected at CE.

#### 5.1.2. Device-Assisted Enteroscopy

For therapeutic purposes, DAE is the gold-standard procedure, with double-balloon enteroscopy preferred over single-balloon enteroscopy [117].

DAE-assisted polypectomy has been widely used for the resection of SB polyps in patients with PJS. To date, hot snare polypectomy and endoscopic mucosal resection (EMR) are the most used techniques [22,134]. Recently, a new technique called endoscopic ischemic polypectomy (EIP) has shown promising results [143].

Some studies reported a progressive decrease in polyp burden, polyp-related complications, and surgery rate in PJS patients undergoing SB surveillance, following the current guidelines’ recommendations [144,145,146,147,148].

In 2019, Perrod et al. reported their experience of enteroscopy resection of SB polyps in 25 patients affected by PJS with detected polyps >1 cm at CE/MRI-E. Overall, enteroscopy resection of 216 SB polyps (median 8.6 polyps per patient, size 6–60 mm) was performed in 50 enteroscopies. Of these, DBE was performed in 33 patients (66%), spiral enteroscopy in 15 (30%), and push enteroscopy in 2 (4%). Complete treatment was achieved in 76%, and intraoperative enteroscopy and surgical resection were required in 4 and 2 patients, respectively. Complications were reported after three DAE procedures (6%): two cases of delayed bleeding requiring blood transfusions after DBE and one case of acute pancreatitis after spiral endoscopy.

Moreover, a recent multicentric retrospective study (23 PJS patients, 46 DAE performed) showed the safety and efficacy of DAE-assisted polypectomy in PJS. A total of 131 polypectomies were performed, with an adverse event rate of 1.5%. Interestingly, a decrease in the rate of laparotomy from 75% to 8% over the study period was observed after index surveillance DAE. Thus, the authors proposed the use of DAE at an interval of 2–3 years from the age of 18 to remove all polyps ≥10 mm in PJS patients with confirmed SB hamartomas, suggesting that DAE surveillance and polypectomy may reduce the rate of laparotomies in this setting [149].

Few data are available on the follow-up of PJS patients after DAE polypectomy, with some authors proposing further follow-up with DAE, while others suggesting a more conservative approach with CE/MRI-E surveillance.

In addition, DAE has been reported in the emergency setting to reduce intussusception due to PJS polyps, before small bowel necrosis occurs, possibly avoiding the need for intestinal resection. After the reduction by DBE, elective SB polypectomy by DAE can be successfully performed [150,151,152].

### 5.2. Insights into Clinical Practice

**Figure 5:**
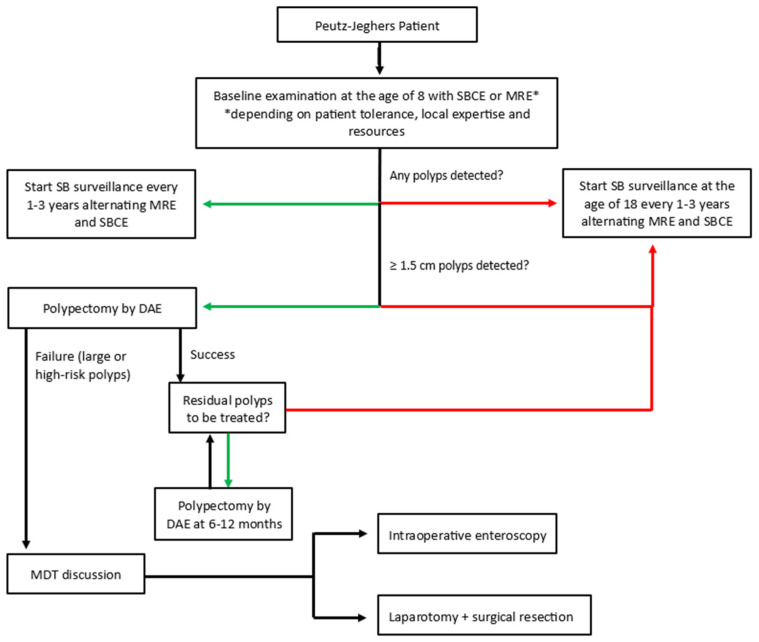
Schematic representation of a proposed protocol for SB surveillance in PJS. Green arrow = yes, red arrow = no. * depending on patient tolerance, local expertise and resources.

PJS is a hereditary disease that significantly reduces the quality of life of patients. Based on the current literature, either CE or MRI-E is recommended for SB surveillance. Currently, local expertise and test availability combined with patient preference determine which test is used preferentially for initial screening. However, in our opinion, a protocol that involves the alternating use of MRI-E and CE at 1–3 years (as suggested by European guidelines) could optimize SB surveillance in patients with PJS, improving the diagnostic accuracy for polyps and patient satisfaction. Figure 5 represents a schematic representation of a proposed protocol for SB surveillance in PJS.

After the identification at non-invasive SB surveillance of significant polyps ≥15 mm, enteroscopy-assisted resection should be proposed, considering DAE as the first-line procedure. The ultimate goal of SB surveillance and treatment in PJS patients is to reduce the risk of adenoumatous transformation and prevent the growth of large polyps increasing the risk of intussusception/obstruction. After polypectomy, we suggest scheduling the follow-up based on the number and size of polyps observed and the presence of residual polyps to be treated. In the presence of fewer than 10 polyps smaller than 10 mm, non-invasive follow-up should be considered, while in cases of patients who have a total of more than 20 polyps resected or a maximum diameter of a polyp larger than 15 mm, a further DAE procedure should be scheduled at 1 year. However, a personalized approach on a case-by-case basis is required.

## 6. Other Inherited Digestive Cancer Syndromes

In other inherited polyposis syndromes, due to the low incidence of these diseases and the difficulties in the evaluation of the small bowel, the epidemiological data on small bowel cancers are scanty and incomplete; therefore, no general recommendations can be made. However, with the widespread adoption of new diagnostic techniques and the increased knowledge of these hereditary syndromes, we expect to achieve a better understanding of the natural history, risk factors, and optimal surveillance strategies for SB cancers in these conditions.

### 6.1. Constitutional Biallelic Mismatch Repair Deficiency Syndrome

Constitutional Biallelic Mismatch Repair Deficiency Syndrome (CMMRD) is a rare autosomal recessive genetic disorder caused by mutations in both copies of mismatch repair (MMR) genes, such as *MLH1*, *MSH2*, *MSH6*, and *PMS2*. These genes are essential for DNA repair, and their dysfunction leads to genomic instability and a high risk of developing cancers. These mutations are particularly associated with gastrointestinal tumors, brain malignancies, and hematological cancers, with typical onset during childhood and adolescence [153]. According to data from small case series and reports, the prevalence of small bowel cancer in individuals with CMMRD ranges from 10% to 16%. The median age of diagnosis is 28 years, with a range from 11 to 42 years. Moreover, they have been reported to occur along all segments of the small bowel [154,155]. Given the high incidence of small intestinal malignancies in these patients, the International CMMRD Consortium and the European Consortium for the Care of CMMRD patients currently suggest annual upper endoscopy and CE starting, respectively, at ages 8 and 10 [156,157]. However, the validity of these recommendations has not yet been thoroughly evaluated, and prospective studies assessing the clinical impact of CE are needed. A recent report from the International CMMRD Consortium highlights that CE can be both useful and feasible. However, it also emphasizes that lesions are often located in the duodenum and may be missed on CE, underscoring the importance of concurrent surveillance with other endoscopic techniques, such as push enteroscopy, with careful inspection of the ampullary region [158].

### 6.2. MUTYH-Associated Polyposis

MUTYH-associated polyposis (MAP) is an autosomal recessive hereditary cancer syndrome and is the second most common cause of adenomatous polyposis syndrome, accounting for approximately 7% of patients with an adenomatous polyposis phenotype [159]. MAP is caused by biallelic pathogenic variants (homozygous or compound heterozygous variants) in MUTYH, which encodes a glycosylase of the DNA base excision repair system [160]. 

Patients with MAP are at increased risk of developing extra-colonic malignancies, including non-melanoma skin cancer, duodenal cancer, ovarian and endometrial cancer, or bladder cancer, among others [161,162].

In MUTYH-associated polyposis (MAP), small bowel surveillance is indicated only in the proximal tract, given the increased risk of duodenal polyposis and cancer. In fact, although duodenal polyposis occurs less frequently in MAP than in FAP (14–34% compared to 65–90%, respectively), and typically develops at a later age, the risk of progression to duodenal cancer is comparable in both conditions [128,163,164]. In contrast, polyps distal to the ligament of Treitz have been rarely reported.

Both American and European current guidelines recommend upper endoscopy, including duodenoscopy with thorough inspection of the duodenum and ampullary site starting, respectively, at age 25–30 and 35 years [47,138].

However, recent retrospective studies suggest that Spigelman staging is not a reliable predictor of cancer risk in MAP because it fails to identify patients at risk of duodenal cancer [164]. Based on this evidence, the updated European guideline does not recommend the use of Spigelman staging to determine the upper-GI surveillance interval in MAP [48]. In addition, in this setting small bowel adenocarcinomas may involve the distal portion of the duodenum and high-grade dysplasia may also be present in small adenomas. For these reasons, a recent update of European guidelines recommends polypectomy regardless of polyp size or Spiegelman staging [48]. Push enteroscopy, side-view upper endoscopy and proactive endoscopic intervention could be considered to better prevent small bowel cancer in MAP [128,163].

### 6.3. Juvenile Polyposis Syndrome

Juvenile Polyposis Syndrome (JPS) is characterized by multiple juvenile hamartomatous polyps in the gastrointestinal tract [165]. JPS is caused by *SMAD4* or *BMPR1A* germline variants. Both genes are tumor suppressor genes encoding for signaling proteins that suppress cell proliferation through the transforming growth factor (TGF)-β pathway [166,167]. Approximately 75% of cases are inherited in an autosomal dominant manner, whereas the rest are sporadic cases [168].

JPS is classified into three categories according to phenotypic features of polyp distributions: generalized juvenile polyposis, juvenile polyposis coli, and juvenile polyposis of the stomach [165].

Involvement of the small bowel in JPS is uncommon and primarily affects the duodenum, with a 29% prevalence of duodenal polyps in individuals with *SMAD4* mutations, according to Wain et al. [169]. Since routine imaging for small bowel polyps in JPS is lacking, the true incidence remains unclear. However, distal duodenal involvement appears rare, and no cases of carcinoma in the jejunum or ileum have been reported to date [82,170]. Consequently, esophagogastroduodenoscopy appears sufficient for upper-GI surveillance and current guidelines do not recommend small bowel surveillance in asymptomatic JPS patients. CE, CT enterography, and eventually, enteroscopy appear reasonable in patients with unexplained anemia, protein-losing enteropathy, or other small bowel symptoms [138,171]. Notice that both small bowel polyps and angioectasia can be present, especially in patients with *SMAD4* mutations and hereditary hemorrhagic telangiectasia, and they can be identified through capsule endoscopy [170].

### 6.4. Cowden Syndrome

Cowden syndrome is a genetic syndrome caused by a germline pathological variant in the *PTEN* and is inherited in an autosomal-dominant manner. It is characterized by the presence of multiple hamartomatous lesions in various organs, including the skin, mucosa, breast, thyroid gland, endometrium, gastrointestinal tract, and brain. Patients with Cowden syndrome have a high risk of developing different types of malignancy, such as breast cancer, thyroid cancer, endometrial cancer, colorectal cancer, and renal cell carcinoma; hence, appropriate surveillance is necessary [168].

Small bowel polyps may occur in these patients, especially in the duodenum and jejunum. Polyp histology includes hamartomas, hyperplastic polyps, ganglioneuromas, adenomas, and inflammatory polyps. These polyps are usually small (2–5 mm) and their color is similar to that of the surrounding mucosa. Given these features, it could be difficult to detect them with conventional techniques such as double-contrast X-ray study [172]. However, CE has proven to be useful for detecting these polyps [172]. There are some case reports of duodenal adenocarcinoma and of jejunal gastrointestinal stromal tumors [173,174], but these have not been found to be common in larger case series. So, at present, routine screening is not recommended, and endoscopic evaluations should be reserved for specific patients based on their symptoms [47,138].

### 6.5. Serrated/Hyperplastic Polyposis Syndrome

Serrated polyposis syndrome (SPS) is characterized by the presence of multiple and/or large serrated polyps in the colorectum and by an increased risk of CRC [175]. Besides a small proportion of cases caused by germline mutations in *RNF43*, no clear genetic cause has been identified [176]. Both epigenetic and environmental factors, especially smoking, have been related to this syndrome, but the etiology of SPS remains uncertain and diagnosis is based on endoscopic criteria [176]. Based on the current evidence, SB is not considered a target organ in serrated/hyperplastic polyposis syndrome and there is no evidence to support extracolonic cancer surveillance at this time [47,138].

## 7. Future Perspectives

Patients with hereditary colorectal cancer syndromes, particularly LS, FAP, and PJS, have an elevated risk of SBC, necessitating targeted surveillance. FAP is associated with duodenal adenomas, with an 18% lifetime risk of duodenal cancer, guiding surveillance through the Spigelman classification and periodic upper endoscopy, while jejunal and ileal polyps remain less studied, As Kasper et al. suggested in a recent review for the management of desmoid tumors, which is another major challenge in FAP individuals, that a prospective study implementing radiological abdominal surveillance (e.g., a magnetic resonance enterography) may tackle the uncertainty about the correct incidence of both SBC and desmoid tumors [177]. LS patients, particularly those with *MLH1* and *MSH2* mutations, have a 4.2% risk of SBC, primarily in the duodenum and jejunum, making extended duodenoscopy preferable to routine CE. Recently, Moller et al. compiled a prospective LS database (PLSD) which revealed that the risk of cumulative incidence of SBC in male patients at 75 years of age with *MLH1* and *MSH2* mutations is similar to that of gastric cancer [120]. However, no surveillance is currently applied for SBC, while upper endoscopy is recommended every 2–3 years in all the international guidelines. Thanks to this new evidence, it will be possible to redesign the surveillance for the sub-cohort of patients at high SBC risk, proposing a CE every 2–4 years. In PJS, hamartomatous polyps require regular monitoring and polypectomy to prevent complications. Advancements in molecular profiling, imaging, and artificial intelligence (AI) are refining surveillance strategies. Genetic risk stratification would enhance patient selection, reducing unnecessary procedures while improving early detection. AI-assisted analysis in CE and MRE improves lesion detection accuracy and workflow efficiency. Specifically, AI and deep learning have profoundly revolutionized the medical imaging. However, challenges remain in training machine learning models for rare diseases such as SBC. Despite these difficulties, the growing availability of multicentric studies and access to medical image storage offer promising solutions to overcome these limitations. Liquid biopsy and biomarker research offer potential for non-invasive identification of precancerous lesions. Multidisciplinary teams in tertiary centers, including gastroenterologists, geneticists, radiologists, and oncologists, are essential for optimizing surveillance. Genetic counseling plays a pivotal role in assessing familial risk, guiding surveillance timing, and informing risk-reduction strategies. Centralized care ensures standardized protocols, reduces unnecessary interventions, and improves patient outcomes. The surveillance burden remains a challenge, but early detection reduces emergency surgeries and late-stage cancer treatments, justifying specialized care investment. Structured systems for referral to tertiary centers optimize healthcare resources by concentrating expertise and streamlining management. In conclusion, effective small bowel surveillance requires a multidisciplinary approach involving genetic counseling and specialized endoscopic techniques. Centralizing care in tertiary centers improves clinical outcomes and resource allocation. Future research should integrate AI, biomarkers, and cost-effective strategies to refine surveillance and reduce disparities in hereditary gastrointestinal cancer care. 

## Figures and Tables

**Table 1:** Main characteristic of currently available enteroscopy techniques [16,31,32,33].

	Capsule Endoscopy (CE)	Device-Assisted Enteroscopy DAE			Intraoperative Enteroscopy (IOE)
		Double-Balloon Enteroscopy (DBE)	Single-Balloon Enteroscopy (SBE)	Spiral Enteroscopy SE	
**Procedure Type**	Non invasive	Invasive			Invasive, during surgery
**Setting**	Outpatient	Inpatient			Inpatient
**Sedation needed**	No	Yes			Anaesthesia
**Bowel preparation**	Yes	Yes			Yes
**Radiation exposure**	No	No			No
**Complete enteroscopy**	80–97%	40–60%	15–25%	10–60%	N/A
**Movement**	Peristalsis	Pleating	Pleating	Screw-mechanism	Pleating and manual external manipulation
**Adverse events rate**	Capsule retention (1.2–2%)	Bleeding, perforation (0.3–8%)			Bleeding, perforation (0–5%)
**Lumen inflation**	No	Yes			Yes
**Software analysis**	Yes	No			Yes/No
**Tissue sampling**	No	Yes			Yes
**Therapeutic procedures**	No	Yes			Yes

**Table 2:** Modified Spigelman classification of duodenal polyposis in FAP.

	1 Point	2 Points	3 Points
N. of polyps	1–4	5–20	>20
Polyp size (mm)	1–4	5–10	>10
Histology	Tubular	Tubulovillous	Villous
Dysplasia	Low grade		High grade

Legend FAP, familial adenomatous polyposis. Stage 0, 0 points; stage I, 1 to 4 points; stage II, 5 to 6 points; stage III, 7 to 8 points; stage IV, 9 to 12 points.

**Table 3:** Duodenal cancer risk in FAP according to Spigelman stage.

Stage	Duodenal Cancer Risk
Spigelman stage 0	Lifetime risk = 0% 10-year risk = 0%
Spigelman stage I	Lifetime risk = 0% 10-year risk = 0%
Spigelman stage II	Lifetime risk = 12% 10-year risk = 2%
Spigelman stage III	Lifetime risk = 13% 10-year risk = 2%
Spigelman stage IV	Lifetime risk = 33% 10-year risk = 36%

**Table 4:** Capsule endoscopy outcome data in LS patients reported in the literature.

Article	Type of Study	N Patients	CE Yield	Cecum Visualization	Adverse Events	Diagnosis
Perrod G et al., 2020 [110]	Observational, retrospective single-center	135	4.4% 6/135	97.7% 132/135	0	3 adenoma 3 adenocarcinoma
Saurin JC et al., 2010 [108]	Prospective, multicenter	35	20% 7/35	91.2% 31/34	0	1 adenoma 1 adenocarcinoma
Haanstra J. et al., 2015 [109]	Prospective, multicenter	200	8.5% 17/200	95% 190/200	0	1 carcinoma 1 adenoma
Haanstra J. et al., 2017 [118]	Prospective, multicenter	155	10% 17/155	86.5% 134/155	0	0 SBN

**Table 5:** Comparison of American and European guidelines for small bowel surveillance in Peutz–Jeghers syndrome.

Scientific Society	Procedure/Method/ Technique	Baseline Examination	Starting Age of Surveillance	Interval Timing
American College of Gastroenterology	CE CT Enterography is accurate at detecting small bowel polyps ≥ 1 cm, but repeated X-ray exposure is problematic.	Age 8 years	If no polyps are found at baseline examination, repeat at age 18	Every 3 years or earlier if symptoms occur
American Gastroenterological Association	CE or MRI-E	Between ages 8–10 years or earlier if the patient is symptomatic	If no polyps are found at baseline examination, surveillance should resume at age 18	Every 2–3 years
European Society of Gastrointestinal Endoscopy	CE or MRI-E	Age of 8 years in asymptomatic individuals		1–3 years based on phenotype
European Hereditary Tumour Group	CE or MRI-E	Age of 8 years in asymptomatic individuals		1–3 years based on phenotype

CE: capsule endoscopy; CT: computed tomography; MRI-E: magnetic resonance enterography.

## Abbreviations

The following abbreviations are used in this manuscript:

FAP	Familial Adenomatous Polyposis
LS	Lynch Syndrome
HNPCC	Hereditary Non-Polyposis Colorectal Cancer
PJS	Peutz-Jeghers syndrome
MAP	MUTYH-associated polyposis
JPS	Juvenile Polyposis Syndrome
SBC	Small Bowel Cancer
CE	Capsule Endoscopy
DAE	Device-Assisted Enteroscopy
DBE	Double-Balloon Enteroscopy
SBE	Single-Balloon Enteroscopy
PE	Push Enteroscopy
AI	Artificial Intelligence
SB	Small Bowel
CMMR-D	Constitutional Mismatch Repair Deficiency
SPS	Serrated Polyposis Syndrome
GI	Gastrointestinal
CT	Computer Tomography
MR	Magnetic Resonance
*APC*	Adenomatous Polyposis Coli
CRC	ColoRectal Cancer
AFAP	Attenuated Familial Adenomatous Polyposis
EGD	Esophagogastroduodenoscopy
EMR	Endoscopic Mucosal Resection
ESD	Endoscopic Submucosal Dissection
EUS	Endoscopic Ultrasound
ERCP	Endoscopic Retrograde Cholangiopancreatography
EP	Endoscopic Papillectomy
MRI-E	Magnetic Resonance Enterography
ASGE	American Society for Gastrointestinal Endoscopy
ESGE	European Society of Gastrointestinal Endoscopy
EHTG	European Hereditary Tumour Group
AGA	American Gastroenterological Association
*MLH1*	MutL Homolog 1
*MSH2*	MutS Homolog 2
*MSH6*	MutS Homolog 6
*PMS2*	PostMeiotic Segregation increased 2
*EPCAM*	Epithelial Cell Adhesion Molecule
MUTHY	mutY DNA glycosylase
*PTEN*	Phosphatase and Tensin homolog
*STK11*	Serine Threonine Kinase 11
*LKB1*	Liver Kinase B1 Protein
*SMAD4*	Small Mother Against Decapentaplegic family member 4
*BMPR1A*	Bone Morphogenetic Protein Receptor type 1A
*RNF43*	Ring Finger Protein 43
